# Bi-Hormonal Endocrine Cell Presence Within the Islets of Langerhans of the Human Pancreas Throughout Life

**DOI:** 10.3390/cells14010034

**Published:** 2025-01-01

**Authors:** Jiwon Hahm, Dawn Kumar, Juan Andres Fernandez Andrade, Edith Arany, David J. Hill

**Affiliations:** 1 Department of Physiology and Pharmacology, Schulich School of Medicine and Dentistry, Western University, London, ON N6A 3K7, Canada; jhahm2@uwo.ca (J.H.); juanfernandez2509@gmail.com (J.A.F.A.); 2 Lawson Health Research Institute, St. Joseph’s Health Care, London, ON N6A 4V2, Canada; dawnniv11@gmail.com (D.K.); earany@uwo.ca (E.A.); 3 Faculty of Science, McMaster University, Hamilton, ON L8S 4L8, Canada; 4 Department of Medicine, Schulich School of Medicine and Dentistry, Western University, London, ON N6A 3K7, Canada

**Keywords:** human, pancreas, islets of Langerhans, bi-hormonal cells, insulin, lifespan

## Abstract

Bi-hormonal islet endocrine cells have been proposed to represent an intermediate state of cellular transdifferentiation, enabling an increase in beta-cell mass in response to severe metabolic stress. Beta-cell plasticity and regenerative capacity are thought to decrease with age. We investigated the ontogeny of bi-hormonal islet endocrine cell populations throughout the human lifespan. Immunofluorescence microscopy was performed for insulin, glucagon, and somatostatin presence on paraffin-embedded sections of pancreata from 20 donors without diabetes aged between 11 days and 79 years of age. The mean proportional presence of glucagon-, insulin-, and somatostatin-immunoreactive cells within islets was 27.5%, 62.1%, and 12.1%, respectively. There was no change in the relative presence of alpha- or beta-cells with advancing age, but delta-cell presence showed a decline with age (R^2^ = 0.59, *p* < 0.001). The most abundant bi-hormonal cell phenotype observed co-stained for glucagon and insulin, representing 3.1 ± 0.3% of all islet cells. Glucagon/somatostatin and insulin/somatostatin bi-hormonal cells were also observed representing 2–3% abundance relative to islet cell number. Glucagon/insulin bi-hormonal cells increased with age (R^2^ = 0.30, *p* < 0.05) whilst insulin/somatostatin (R^2^ = 0.50, *p* < 0.01) and glucagon/somatostatin (R^2^ = 0.35, *p* < 0.05) cells decreased with age of donor. Findings show that bi-hormonal cells are present within human pancreatic islets throughout life, perhaps reflecting an ongoing potential for endocrine cell plasticity.

## 1. Introduction

The regenerative capacity of beta-cells within the pancreatic islets of Langerhans is limited by their extremely low proliferation rate postnatally both in humans and in animal models [1,2]. Similarly, while new beta-cells can originate from endogenous progenitor cells in early life, progenitor abundance declines rapidly after puberty [3]. However, functional beta-cell mass can change dramatically under metabolic stress such as type 2 diabetes, through lineage plasticity of islet endocrine cells. An increased number of glucagon/insulin bi-hormonal cells were observed within islets of donors who were insulin-resistant compared with normoglycemic controls [4] and were postulated to represent an intermediate phenotype in the transdifferentiation of alpha- to beta-cells.

The functional phenotypes of proglucagon vs. insulin gene expression for alpha and beta-cells, respectively, depends on differential transcription factor expression, and experimental alterations in this balance can alter cell lineage fate. Human islets from donors with or without type 2 diabetes demonstrated alpha- to beta-cell transdifferentiation after transplantation into diabetic mice following transfection with PDX1 and MAFA, two transcription factors necessary for the adoption of a beta-cell phenotype, and similar results were found following ablation of a key transcription factor necessary for alpha-cell functionality, ARX [5]. Similarly, the ectopic expression of Pax4, a key defining transcription factor for beta-cells, could induce the conversion of alpha-cells into insulin-producing cells in mice [6], while transfection of human islets from individuals with type 2 diabetes with PAX4 increased the number and function of beta-cells [7]. Mouse models of diabetes associated with extreme beta-cell loss have demonstrated that reprogramming occurs in around 2% of islet alpha-cells to allow transdifferentiation to beta-cells, with an intermediate cell phenotype of both glucagon and insulin expression [8]. Inhibition of the glucagon receptor was also shown to reverse diabetes in type 1 diabetic mice through islet cell transdifferentiation, with around 15% of new beta-cells being derived from alpha-cells in lineage tracking studies [9].

A potential for islet cell trandifferentiation may not be limited to alpha-cells. A bi-hormonal cell phenotype expressing both somatostatin and insulin was found to be an intermediate in the transdifferentiation of delta to beta-cells following beta-cell ablation in a zebrafish model [10]. In young mice subjected to beta-cell ablation, most subsequent regeneration of beta-cells was by transdifferentiation from delta-cells, but this was age-dependent with alpha- to beta-cell transdifferentiation becoming dominant following puberty [11]. However, a pancreatic duct source of transdifferentiation may have persisted into adulthood whereby Ngn3-expressing endocrine progenitors within the pancreatic duct epithelium expressed somatostatin and subsequently transdifferentiated to become beta-cells [12].

While evidence exists for islet cell transdifferentiation as a mechanism to increase beta-cell mass following metabolic stress, it is unclear to what extent a bi-hormonal population exists in the human pancreas under normal glucose homeostasis. Glucagon/insulin bi-hormonal cells were observed in pancreata from non-diabetic human subjects [13], but how this might change with age is not known. Several animal and human studies have reported a relationship between increasing age and a decrease in islet cellular plasticity [14,15], which might be related to bi-hormonal cell abundance. Therefore, here we have investigated the presence of bi-hormonal cells in non-diabetic humans and how this might change with age.

## 2. Materials and Methods

Paraffin-embedded blocks from human pancreata were kindly provided by the University of Chicago and the Department of Pathology and Laboratory Medicine, London Health Sciences Centre, London, Ontario with approval of the Human Research Ethics Board of Western University (#103167), the Institutional Review Board at the University of Chicago, and Lawson Health Research Institute (R-12-501). The donors were ten males and ten females, with ages ranging from 11 days to 79 years (Table 1). The mean age of male donors was greater than that of female donors (45 vs. 25 years).

Samples were selected from cadaveric tissue taken from individuals without known metabolic disease who had trauma-associated deaths. The tissues were harvested under criteria considered to be suitable for organ transplantation. The body mass index of the donors was within the range 18–25 kg/m^2^, and donors had a diversity of ethnic backgrounds including Caucasian, Black, and Hispanic. Pancreata were fixed within 6–12 h of cold ischemia to minimize changes post mortem and paraffin blocks were selected from the mid-pancreas anatomical region in each case. Tissues were sectioned using a microtome at 5 µm thickness, with three replicate sections from each pancreas being collected with >100 µm intervals between each section to ensure that different cell populations were analyzed between replicate sections. Pancreas sections were deparaffinized with xylene and ethanol prior to immunohistochemistry. Epitope retrieval was performed by heating sections in a pressurized chamber (declocker) at 100 °C for 15 min in sodium citrate buffer (10 mM sodium citrate, 0.05% Tween 20, pH 6.0). Non-specific binding was minimized by the application of Sniper universal block (Biocare Medical, Concord, CA, USA) to sections during an 8 min incubation. Glucagon (mouse anti-glucagon 1:2000, Sigma-Aldrich, St. Louis, MO; RRID:AB_259852); insulin (guinea pig anti-insulin 1:20, Thermo Fisher Scientific, Waltham, MA USA; RRID:AB_794668); and somatostatin (rabbit anti-somatostatin 1:100, Thermo Fisher Scientific; RRID:AB_2789834) primary antibodies were applied together to slides, which were then incubated at 4 °C overnight in a humidified chamber. Specific secondary fluorescent antibodies were applied the following day (goat anti-guinea pig, Alexa Fluor 647, RRID:AB_2535867; goat anti-mouse, Alexa Fluor 555, RRID:AB_2633276; and goat anti-rabbit, Alexa Fluor 488, RRID:AB_143165, all at 1:500; Thermo Fisher Scientific) for 90 min before washing and the addition of 4′,6-diamidino-2-phenylindole dihydrochloride (DAPI) (Thermo Fisher Scientific) to counterstain the nuclei. Tissue sections were then mounted with glass coverslips using Fluorescence Mounting Medium (Dako Canada, Burlington, ON, Canada) and sealed with clear nail polish.

Microscopy was performed using a Nikon A1R confocal microscope (Nikon, Minato, Tokyo, Japan) located at the Lawson Research Institute to capture x20 magnification images for image analysis. A z-plane image stack was also captured for islets within each section to confirm that more than one hormone was being visualized within individual cells and to omit instances where overlapping cells had been imaged.

Islet of Langerhans were considered to be structures surrounded by a basement membrane containing more than six endocrine cells staining for either insulin, glucagon, or somatostatin. Quantification of each endocrine cell type was performed using Fiji Image J2 software (https://imagej.net/software/fiji/, 28 November 2024) to quantify islet cells staining for individual or bi-hormonal/tri-hormonal combinations. All islets within a pancreas section were examined with the mean number of islets analyzed per donor being 326 (range 89–495). To quantify total insulin-, glucagon-, or somatostatin-positive cells we used an area analysis macro developed in our laboratory within Fiji Image J2 software. The macro transformed single channel images of insulin, glucagon, and somatostatin signals into binary images to distinguish areas with or without a signal, as described previously by Phansalkar [16]. The area representing a positive signal was measured and corrected for the average area of an individual alpha-, delta-, or beta-cell to estimate the number of each cell type within an islet. Co-staining cells were identified as above and counted manually by assigning the two antigens to be quantified with either red or green filters on the Fiji Image J2 software, where bi-hormonal cells would display an intermediate color.

Data are presented as mean ± SEM or by distribution of individual values with advancing age. The numbers of alpha-, beta-, or delta-cells within islets were found to follow a curvilinear distribution with age and were therefore analyzed by non-linear regression after exponential transformation. The abundance of bi-hormonal cell combinations within islets was found to be linearly distributed with age and was analyzed by linear regression using Prism 10 software (Graphpad Software, Boston, MA, USA). The statistical significance for R^2^ was set at *p* < 0.05.

## 3. Results

### 3.1. Islet Morphology

**Figure 1:**
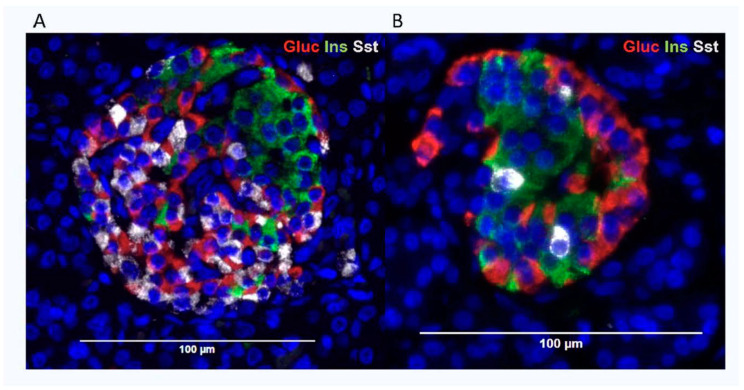
Distribution of alpha-cells (glucagon—red), beta-cells (insulin—green), and delta-cells (somatostatin—white) in representative human pancreatic islets from (**A**) an 11-day-old donor and (**B**) a 52-year-old individual. Cell nuclei are visualized in blue. The bars indicate islet size.

The beta-cells within human islets in this study showed a typical distribution of regional clustering and a sometimes ribbon-like appearance within the islets (Figure 1). No differences in morphology were visually observed with age of donor, and glucagon-containing alpha-cells and somatostatin-containing delta-cells were distributed evenly throughout the islets, interspersed with beta-cells, as described by others [17]. The mean proportional presence of glucagon-, insulin-, and somatostatin-immunoreactive cells within islets was 27.5% (range 40.5–17.4%), 62.1% (range 83.9–25.1%), and 12.1% (range 33.8–2.8%), respectively, demonstrating large variability in relative endocrine cell abundance across the age range. When the proportion of endocrine cells was considered by donor sex the relative abundance of beta-cells was significantly greater in males than in females (male 69.7 ± 5.8%, female 53.7 ± 3.1%, *p* < 0.05), whilst delta-cell abundance was higher in females (male 8.26 ± 0.1%, female 17.8 ± 4.4%, *p* < 0.05). Alpha-cell abundance did not differ with sex (male 27.4 ± 1.6%, female 27.7 ± 3.3%, *p* = 0.92).

**Figure 2:**
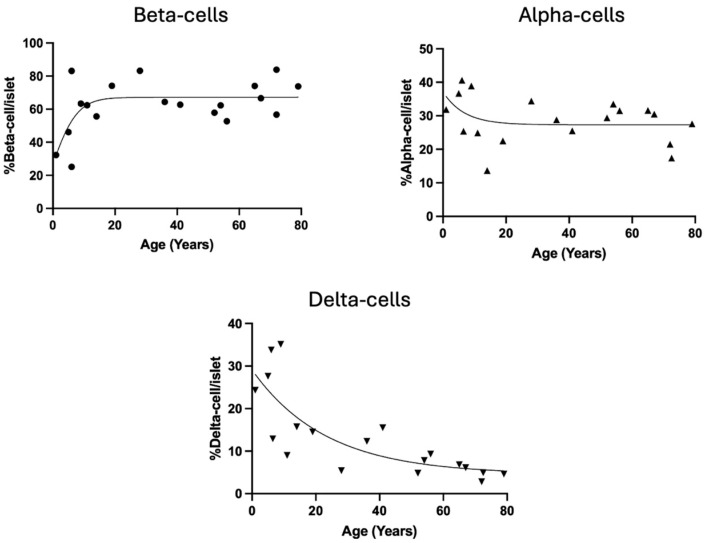
Mean percentage of beta-, alpha-, and delta-cells within islets of Langerhans for individual pancreas donors distributed by donor age. The best-fit line is shown following non-linear regression analysis.

The changes in mean alpha-, beta-, and delta-cell presence per islet with donor age are shown in Figure 2. Each cell type showed a curvilinear distribution with age with beta-cells being relatively less abundant below the age of 20, while alpha- and delta-cells were more abundant at early ages. After age 20, neither beta- nor alpha-cells changed significantly with age, but delta-cells continued to decline significantly with age (R^2^ = 0.59, *p* < 0.001). The slopes of the regression lines did not significantly differ for any of the three cell types when male and female donors were considered separately.

### 3.2. Bi-Hormonal Cell Presence

**Figure 3:**
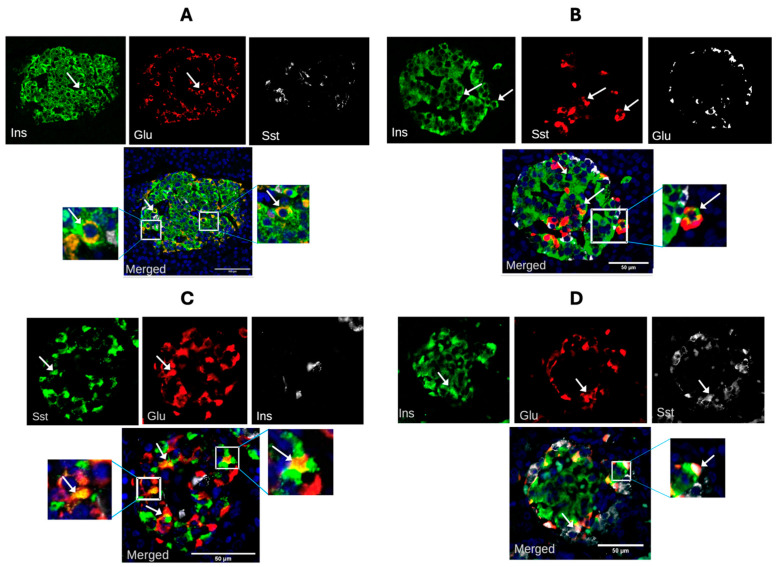
Examples of bi-hormonal endocrine cells in human islets (arrows). Red and green fluorescence filters were assigned to identify cells bi-hormonal for the co-presence (arrows) of insulin (Ins) and glucagon (Glu) ((**A**)—male, 11 years); insulin and somatostatin (Sst) ((**B**)—female, 28 years); or Sst and Glu ((**C**)—female, 5 years), or examples of tri-hormonal cells containing Ins, Glu, and Sst ((**D**)—female, 5 years). Islet size is indicated in each panel by a size bar on the merged image.

We investigated the presence and relative abundance of different islet bi-hormonal cells across the human lifespan for individuals without diabetes or other metabolic disease. Three bi-hormonal endocrine cell phenotypes were observed: cells containing both glucagon and insulin, cells containing insulin and somatostatin, and cells containing glucagon and somatostatin (Figure 3A–C). Tri-hormonal cells containing insulin, glucagon, and somatostatin were also seen with low abundance (Figure 3D).

**Figure 4:**
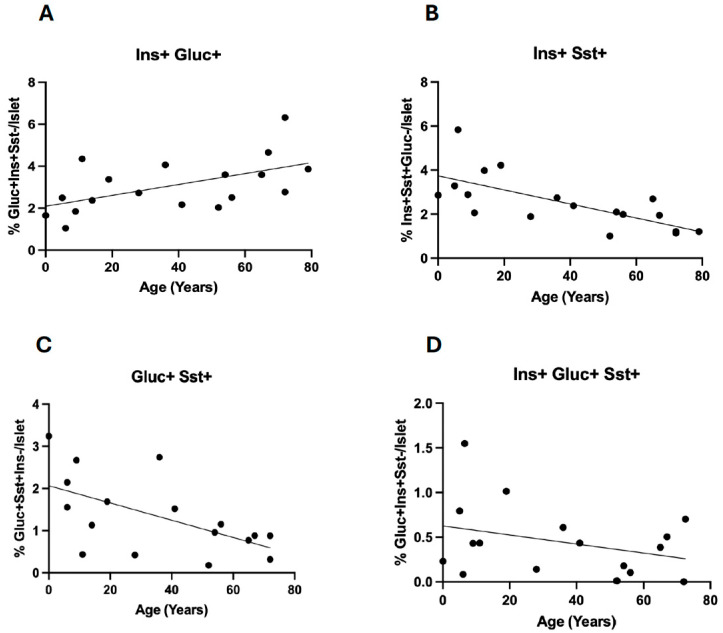
Percentage of multi-hormonal cell phenotypes within islets of Langerhans for individual pancreas donors distributed by donor age and expressed relative to all islet cells ((**A**) insulin/glucagon bi-hormonal cells, (**B**) insulin/somatostatin bi-hormonal cells, (**C**) glucagon/somatostatin bi-hormonal cells, (**D**) insulin/glucagon/somatostatin tri-hormonal cells). Data were analyzed by linear regression and the regression lines are shown.

Bi-hormonal cells were often preferentially located towards the rim of the islets, and this did not alter with age. The most abundant bi-hormonal cell type seen represented a co-localization of glucagon and insulin (3.1 ± 0.3% of total islet cells; range 8.3–1.1%). When separated by sex of donor, insulin/glucagon bi-hormonal cells were significantly more abundant in males (4.0 ± 0.4%) than in females (1.9 ± 0.3%, *p* < 0.001). Linear regression analysis showed a significant positive association of insulin/glucagon bi-hormonal cell presence with donor age (R^2^ = 0.30, *p* < 0.05) (Figure 4A), but this was not influenced by donor sex. Glucagon/somatostatin and insulin/somatostatin bi-hormonal cells each represented 1.7% (range 7.9–1.1%) and 2.5% (range 5.8–0.1%) of total islet cell number, respectively. The abundance of neither type of bi-hormonal cell differed significantly with donor sex. Linear regression analysis showed a negative association of glucagon/somatostatin (R^2^ = 0.35, *p* < 0.05) and insulin/somatostatin (R^2^ = 0.50, *p* < 0.01) cell abundance with donor age (Figure 4B,C). Tri-hormonal cells showed no significant change with donor age (R^2^ = 0.11) (Figure 4D).

## 4. Discussion

The relative presence of the major endocrine cell types within human islets agrees with previous reports, with the ratio of beta-, alpha-, and delta-cells being approximately 60%, 30%, and 10% respectively [18]. However, substantial variation has been reported in the distribution of cell types between adult individuals [19], as also found here. Alpha-cell abundance relative to beta-cells has been reported to increase with age until around 30 years, whilst delta-cell abundance decreases with age [18]. In our data the relative proportions of alpha- vs. beta-cells did not significantly change in adults, although beta-cell proportional presence increased during childhood to reach adult values around the time of puberty. It has been calculated that beta-cell mass increases thirty-fold between birth and adulthood [20], with the highest rates of increase in the first two years of life [14], after which beta-cell proliferation falls to basal values [21]. Whilst beta-cell mass is maintained in later life, insulin secretion capacity may decline since the expression of PDX1, a transcription factor necessary for insulin gene expression, is lower after the age of 60 [22]. Alpha-cell presence was shown to increase with age, although this only occurred after age 70, and with considerable individual variation [23]. Although donors in our study over 70 were few, there was no tendency towards higher alpha-cell abundance. The decline in the proportion of delta-cells with age was pronounced in our study, and since alpha- and beta-cell presence was stable in adults this is likely to indicate a loss of delta-cell number.

We found that bi-hormonal cells containing glucagon/insulin, insulin/somatostatin, or glucagon/somatostatin each represented around 2–3% of total islet endocrine cells in individuals without diabetes or other metabolic diseases. A similar level of insulin-immunoreactive cells, also containing either glucagon or somatostatin, has been reported within human islets and was increased in donors with type 2 diabetes [18,24]. Such bi-hormonal cells first appear in the human fetal pancreas at around 6–8 weeks gestation as endocrine cell clusters but decrease to levels similar to that found here at postnatal ages by 10 weeks’ gestation [25]. This ontogeny is supported by the differentiation of alpha- and beta-cells from human embryonic stem cells where insulin/glucagon bi-hormonal cells appear coincident with the appearance of the endocrine cell populations in vitro [26]. Pancreata from human subjects with insulin resistance showed a 2–3-fold increase in insulin/glucagon bi-hormonal cells compared with individuals without insulin resistance, especially within larger islets [4]. Whilst this may represent alpha- to beta-cell transdifferentiation to increase insulin secretion capacity and counteract insulin resistance, it might also represent a dedifferentiation of beta-cells to an alpha-cell phenotype as a result of metabolic stress, as reported to occur on overt type 2 diabetes [27]. However, no correlation was found between bi-hormonal cell abundance and basal or first-phase insulin secretion, or total insulin secretion during a hyperglycemic clamp, suggesting that bi-hormonal cell presence was not directly linked to insulin demand [4].

In the present study we used immunofluorescence microscopy to colocalize both glucagon and insulin peptides to individual islet cells. However, bi-hormonal cell presence identified by microscopy is supported by published transcriptomic data. Using both single cell RNA sequencing (scRNA-seq) and single cell nucleus RNA sequencing, Kang et al. [28] identified five different sub-clusters of glucagon-expressing cells within isolated islets from pancreata of non-diabetic humans. One sub-cluster co-expressed insulin and could transdifferentiate into either glucagon or insulin mono-hormonal cells. However, in human islet grafts harvested in vivo, alpha-cells showed unidirectional plasticity towards beta-cells only. Genes expressed specifically during alpha- to beta-cell transition included ZNF385D; TRPM3; CASR; MEG3; and HDAC9, variously representing calcium-channel function, long non-coding RNAs, DNA-binding proteins, and a histone deacetylase. This expression profile would support a transitional cell phenotype undergoing epigenetic and functional remodeling. Saikia et al. [29] used scRNA-seq to identify four distinct alpha-cell sub-clusters, of which one expressed both insulin and glucagon, while Elgamal et al. [30] identified a sub-cluster in isolated human islets that co-expressed insulin and glucagon. Single-cell RNA-seq has also been applied within cultured human pancreatic slices from non-diabetic subjects [31]. Multiple sub-clusters of insulin-expressing cells were identified, of which one co-expressed glucagon as well as TMSB10, a gene associated with cytoskeletal reorganization and cell motility. However, transcriptomic analysis should perhaps not be used as a phenotypic tool in isolation, since dispersed human alpha-cells contained a sub-population with high insulin mRNA content, but without translation to insulin [32]. Collectively these reports suggest that there is a ready capacity of sub-populations of alpha-cells to transdifferentiate partially or fully into insulin-expressing cells.

Despite the available cohort size being relatively small we found that the proportional presence of beta-cells within islets was greater, and that of delta-cells lower, in males compared to females, suggesting that the presence of estrogen in pre-menopausal women did not offer an advantage to maintaining beta-cell mass. Similarly, the abundance of glucagon/insulin bi-hormonal cells was greater in males than in females. However, caution must be applied in drawing conclusions since the mean age of females in the present study was twenty years younger than that of males, and glucagon/insulin bi-hormonal cell presence significantly increased with age. Toledo et al. [18] previously reported that alpha-, beta-, or delta-cell relative abundance within islets did not significantly differ between sexes.

Human beta-cells are highly heterogeneous in phenotype and function within a single islet or pancreas [33]. A resident population of progenitor beta-cells has been described in both humans and rodents that express insulin but are poorly glucose-responsive due to a low expression of the GLUT-2 glucose transporter [34,35]. They are present throughout life but decrease in abundance with age [3]. The possibility that these are equivalent to bi-hormonal cells was ruled out, at least in a mouse model of diabetes, as they are transcriptionally distinct by scRNA-seq [36]. Similarly, hub beta-cells that regionally coordinate intracellular rises in calcium and first-phase insulin release within islets, and are transcriptionally distinct, were shown not to be bi-hormonal [37].

While substantial anatomical diversity exists between human islets within a single pancreas, sub-islet anatomical clusters occur, each possessing a core of beta-cells surrounded by alpha- and delta-cells [38]. This allows for extensive paracrine interactions between beta-, alpha-, and delta-cells in the fine control of nutrient-stimulated insulin release. However, we observed that bi-hormonal cells were often seen within the rim of islets, which has also been proposed as the location of resident beta-cell progenitors in both rodent and human islets [39,40]. It is possible, therefore, that this also represents a preferred location for endocrine cell transdifferentiation. Beta-cell neogenesis from progenitors and the presence of bi-hormonal cells may represent facets of the same pathway if bi-hormonal expression were to represent an intermediate phenotype.

We hypothesized that if bi-hormonal cells were linked to beta-cell regenerative capacity, then their proportional presence might decrease with age. While this was true for insulin/somatostatin and glucagon/somatostatin bi-hormonal cells there was a positive association between glucagon/insulin cell abundance and donor age. Since glucose tolerance decreases with age [41], glucagon/insulin bi-hormonal cell presence may represent an ontological mechanism to replenish insulin availability with an increasing demand during aging. While alpha- to beta-cell transdifferentiation has been observed in humans with insulin resistance [4], alpha- to delta-cell transdifferentiation has not been described previously. However, in a mouse model, around 15% of delta-cells were found to express mRNA for an additional hormone to somatostatin, including glucagon, insulin, and pancreatic polypeptide [42]. While we were unable to meaningfully compare changes with age of bi-hormonal cells in males versus females, it has previously been published that these did not differ [18].

In summary, we found a low presence of bi-hormonal cells throughout life within human pancreata of non-diabetic donors. Recently, similar findings were reported by Lenz et al. [43] using single-cell Western blot analysis of human islets. If these cells are capable of transdifferentiation to yield new beta-cells during metabolic stress, then they could represent a strategic reserve of beta-cell progenitors available for therapeutic interventions. Alpha- to beta-cell transdifferentiation was enhanced in human cells by GLP-1 [29] and in animal models through administration of GABA [44], the latter causing a downregulation of the transcription factor *Arx* in alpha-cells. Other drugs used to treat diabetes, such as the SGLT2 inhibitor, dapagliflozin, also promoted alpha- to beta-cell transdifferentiation in diabetic mice [45]. Studies with isolated human islets viably maintained under organoid microfluidic conditions could allow additional therapeutic investigation.

## Figures and Tables

**Table 1:** Age of pancreas donors (years).

Male	6	11	19	36	50	54	56	65	72	79
Female	Neonate	5	6	9	14	20	28	41	52	72

## Data Availability

Original data can be made available upon request to the communicating author. These data form part of an M.Sc. thesis submitted for examination at Western University by J.H.
